# Evolution of T-cell fitness through AML progression: enhanced bispecific T-cell engager-mediated function of bone marrow T cells at remission compared to initial diagnosis and relapse

**DOI:** 10.1038/s41375-024-02387-4

**Published:** 2024-08-22

**Authors:** Maryam Kazerani, Anetta Marcinek, Nora Philipp, Bettina Brauchle, Jonathan Jonas Taylor, Helena Domínguez Moreno, Andrea Terrasi, Benjamin Tast, Lisa Rohrbacher, Yingshuai Wang, Maximilian Warm, Alica-Joana Emhardt, Giulia Magno, Karsten Spiekermann, Tobias Herold, Tobias Straub, Sebastian Theurich, Gunnar Schotta, Roman Kischel, Veit L. Bücklein, Marion Subklewe

**Affiliations:** 1 Laboratory for Translational Cancer Immunology, LMU Gene Center, Munich, Germany; 2 Department of Medicine III, University Hospital, LMU Munich, Munich, Germany; 3 Division of Molecular Biology, Biomedical Center, Faculty of Medicine, LMU Munich, Martinsried, Germany; 4 Experimental Leukemia and Lymphoma Research (ELLF), Department of Internal Medicine III, University Hospital, LMU Munich, Munich, Germany; 5 German Cancer Consortium (DKTK) and German Cancer Research Center (DKFZ), Heidelberg, Germany; 6 Bioinformatics Unit, Biomedical Center, LMU Munich, Martinsried, Germany; 7 Amgen Research (Munich) GmbH, Munich, Germany; 8 AMGEN Inc., Thousand Oaks, CA USA

**Keywords:** Immunotherapy, Acute myeloid leukaemia, T cells

## To the Editor:

In acute myeloid leukemia (AML), the anti-leukemic potential of allogeneic hematopoietic stem cell transplantation (allo-HSCT) and post-transplant donor lymphocyte infusion (DLI) hinges on the activity of T cells [1]. Bispecific antibodies, including bispecific T-cell-engager (BiTE^®^) molecules, redirect endogenous T cells against neoplastic cells for eradication by CD3-dependent T-cell activation. In B-lymphoid malignancies, high clinical efficacy has led to the approval of several T-cell-recruiting constructs [2, 3]. In AML, several bispecific antibodies have been developed and have shown strong preclinical efficacy [4, 5]. However, albeit early-phase I/II clinical trials in heavily pre-treated patients with R/R AML have yielded promising, dose-dependent results, sustained responses were not observed [6–8].

We hypothesize that T-cell dysfunction contributes to BiTE resistance and a lack of long-term responses in AML. Evidence for the relevance of T-cell fitness to BiTE-mediated activity is derived from patients with B-cell precursor acute lymphoblastic leukemia in whom a predominance of T cells with an exhausted phenotype was associated with failure of blinatumomab treatment [9]. Additionally, transcriptional profiles associated with T-cell dysfunction were found in nonresponding patients [10]. Further evidence of an association between T-cell fitness and BiTE activity was found in a preclinical model of T-cell exhaustion after continuous BiTE exposure [11].

So far, attempts to characterize T-cell phenotype and function in AML patients have yielded variable and sometimes contradictory results. Studies suggest that BM T cells in contrast to peripheral blood T cells better reflect the immune state and are the main mediators of BiTE-mediated cytotoxicity [12, 13]. Hence, characterizing BM T cells at different time points during the course of the disease might help to guide the optimal clinical application of T-cell-based immunotherapies in AML.

**Fig. 1:**
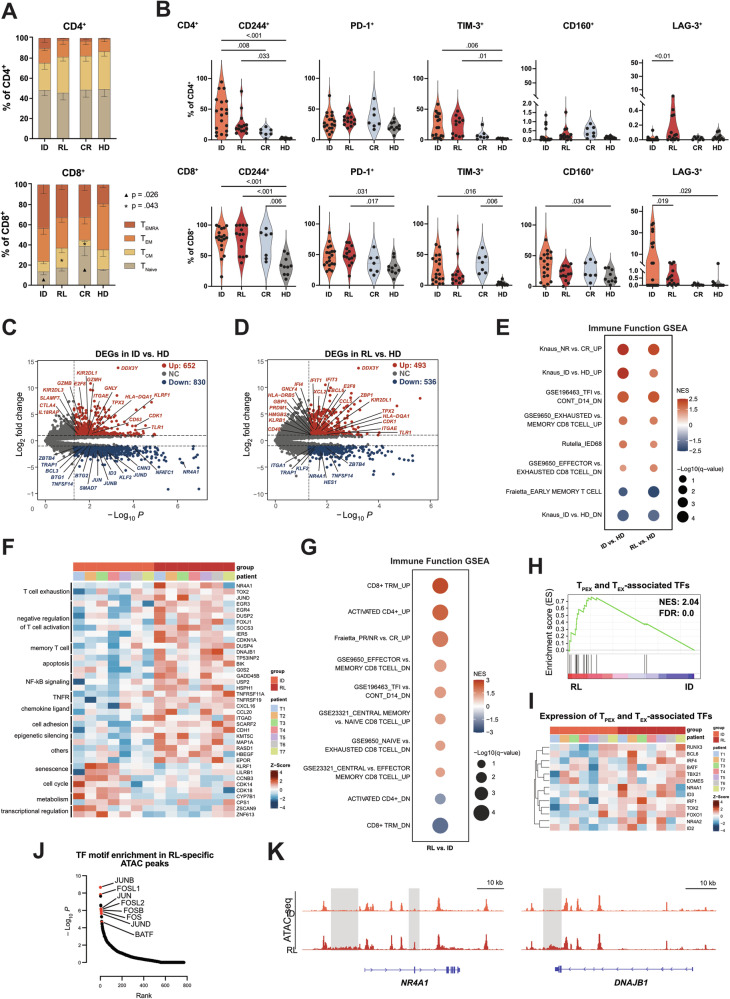
Bone marrow T cells at the time of ID and RL display a phenotypic and transcriptional profile of dysfunction. **A** Proportions of naive (T_Naive_, CD45RA^+^CCR7^+^), central memory (T_CM_, CD45RA^−^CCR7^+^), effector memory (T_EM_, CD45RA^−^CCR7^−^), and terminal effector (T_EMRA_, CD45RA^+^CCR7^−^) T cells within the CD4^+^, and CD8^+^ compartments. **B** Frequency of BM CD4^+^ (top row) and CD8^+^ (bottom row) T cells positive for inhibitory receptors at ID (*n* = 19), RL (*n* = 14), CR (*n* = 7), and in HDs (*n* = 10). **C** Volcano plot of DEGs at ID (*n* = 7) vs. HDs (*n* = 2). Significantly upregulated (red) and downregulated (blue) genes at ID are highlighted (log_2_FC > 1 or < −1; *P* < 0.05). Selected genes are labeled. **D** Volcano plot of DEGs at RL (*n* = 7) vs. HDs (*n* = 2). **E** GSEA for gene sets associated with immune function using published gene sets derived from MSigDB or custom gene sets. GSEA statistics are provided in Supplementary Table 3. **F** Heatmap demonstrating selected DEGs at RL compared to ID. **G** GSEA in RL vs. ID T cells for gene sets associated with T-cell populations and immune function from MSigDB and published data sets (details on the gene sets are provided in the Supplementary Methods). GSEA statistics are included in Supplementary Table 6. **H** GSEA in RL vs. ID T cells for TFs related to T_PEX_ and T_EX_. **I** Heatmap showing the expression of top hits, from the analysis in panel H, in ID and RL patients. **J** TF motifs enriched in RL-specific ATAC peaks. Significant motifs are labeled and highlighted in red. **K** ATAC-seq tracks of selected genes significantly upregulated in RL vs. ID T cells. BM bone marrow, CR complete remission, DEG differentially expressed gene, GSEA gene set enrichment analysis, HD healthy donor, ID initial diagnosis, RL relapse, TF transcription factor. All plots represent the mean ± SEM. One-way ANOVA was used to calculate *P* values.

We assessed BM T cells of AML patients at initial diagnosis (ID), complete remission (CR), and first relapse (RL). All AML samples were allo-HSCT naive, and age-matched HD samples served as a control cohort (Supplementary Table 1). The percentage of BM CD3^+^ T cells was lower at ID and RL compared to time of CR and in HD samples (Supplementary Fig. S1A–C). Of the CD3^+^ T-cell sub-populations, the most significant changes in the T-cell differentiation states were observed in CD8^+^ T cells (Fig. 1A, Supplementary Fig. S1D, E). Terminally differentiated effector cells (T_EMRA_) were the most abundant population at ID compared to other time points. A higher proportion of central memory (T_CM_) cells was apparent at RL compared to ID and CR. By contrast, a higher percentage of naive T cells (T_Naive_) was found at CR than at ID and RL (Fig. 1A).

We next measured the expression of inhibitory receptors within the CD4^+^ and CD8^+^ T cell compartments during AML progression and compared them to HD T cells. A significantly higher proportion of CD244 and TIM-3 expressing cells were detected for both CD4^+^ and CD8^+^ patient T cells. Next, we observed a higher percentage of PD-1^+^ and LAG-3^+^ cells on CD8^+^ patient T cells relative to HDs. In addition, CD8^+^ T cells at ID showed a higher frequency of CD160^+^ cells compared to cells from HDs. Within the CD4^+^ T-cell compartment, we observed a lower proportion of LAG-3^+^ cells at ID compared to RL. In summary, AML patients showed significantly higher expression of exhaustion-associated inhibitory receptors compared to HDs (Fig. 1B, Supplementary Fig. S1F).

To characterize the transcriptional program of AML T cells, we performed longitudinal RNA-seq analysis of sorted BM CD3^+^ T cells from paired ID–RL samples (*n* = 7) and HDs (*n* = 2). We first compared the transcriptional profiles of T cells at both ID and RL to those of HDs and identified 1482 and 1029 differentially expressed genes (DEGs; log_2_FC > 1 or < −1, *P* < 0.05; Supplementary Table 3; Fig. 1C, D, Supplementary Fig. S2A–D), respectively. We observed upregulation of both stimulation as well as dysfunction-associated genes in ID and RL compared to HDs (ID: *CD63*, *GZMH, IL18RAP*, *GZMB*, *CTLA4*; RL: *BLIMP-1, CCL5*, *CD48*, *KLRB1*; both ID and RL: *GNLY, TLR1*). This finding was confirmed by gene set enrichment analysis (GSEA) using published gene sets (Supplementary Methods). Moreover, BM T cells at ID vs. HDs significantly expressed senescence-associated genes like *KLRF1*, and the inhibitory KIRs (*KIR2DL3* and *KIR2DL1*). Notably, the immune effector dysfunction score (IED68) [14] demonstrated significant positive enrichment in ID vs. HD T cells in line with higher expression of senescence-associated markers at this time point (Fig. 1E, Supplementary Fig. S2E–G, Supplementary Table 4).

To elucidate the longitudinal transcriptional changes occurring in patients’ T cells between ID and RL, we compared T cells from AML patients at RL (*n* = 7) to their matched ID counterparts. Differential gene expression analysis (log_2_FC > 1 or < −1, *P* < 0.05; Supplementary Table 5) revealed high expression of senescence markers (*KLRF1*, *LILRB1*) at ID vs. RL and genes related to memory T cells (*DUSP4*, *DNAJB1*) and exhaustion (*NR4A1*, *TOX2*, *JUND*) at RL vs. ID (Fig. 1F, Supplementary Fig. S3A). GSEA indicated that pathways associated with senescence (oxidative phosphorylation and protein secretion) were enriched in ID T cells, whereas RL T cells showed enrichment for T_CM_ and tissue-resident memory T-cell (T_RM_) signatures, as well as pathways implicated in T-cell exhaustion (Fig. 1G, Supplementary Fig. S3B; Supplementary Table 6). Accordingly, expression of core TFs associated with progenitor exhausted (T_PEX_) and terminally exhausted T cells (T_EX_) was significantly enhanced in RL but not ID T cells (Fig. 1H and I, Supplementary Fig. S3C, Supplementary Table 7).

We next looked at active regulatory regions in ID vs. RL T cells. ATAC-seq performed in paired ID and RL T cells identified 1294 differential ATAC peaks (log_2_FC > 1, *P* < 0.01). The principal component analysis separated ID and RL T cells (Supplementary Fig. S3D–F). Notably, accessible regions in RL (RL-specific ATAC peaks) corresponded to changes in TF activity. TF motif analysis on these regions revealed enrichment for AP1 family TF-binding motifs (Fig. 1J, Supplementary Table 8). These findings were in line with higher expression of AP-1 and IRF family members in RL vs. ID T cells (Supplementary Fig. S3G). Furthermore, RL-specific ATAC peaks included the exhaustion-associated gene NR4A1 and the memory-associated gene DNAJB1, which were both transcriptionally upregulated in RL compared to ID T cells (Fig. 1F, K). For regions with decreased accessibility in RL T cells, we did not observe a clear pattern connected to gene expression. Together, these data demonstrate that T cells in AML bear different states of T-cell dysfunction, with senescence appearing to be more prominent at ID, whereas RL T cells exhibit a profile of exhaustion.

**Fig. 2:**
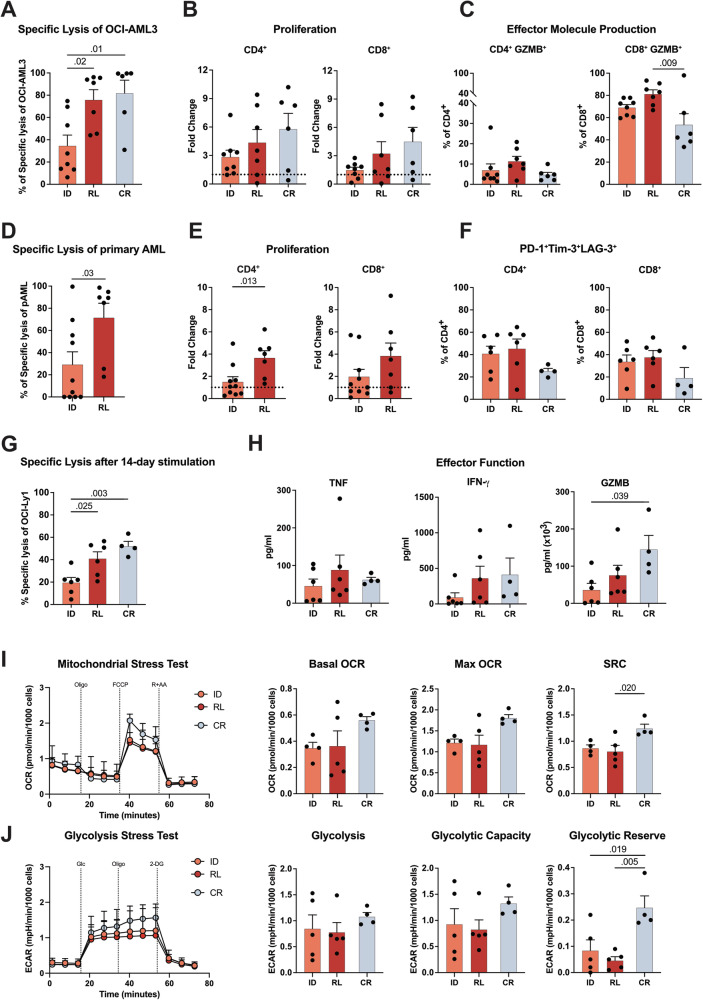
ID T cells display lower BiTE-mediated cytotoxicity compared to RL, but both have impaired metabolic fitness after continuous stimulation. **A** AMG 330-mediated cytotoxicity of T cells sampled at ID (*n* = 8), RL (*n* = 7), and CR (*n* = 6) on day 5 against OCI-AML3 cells relative to cBiTE (concentration AMG 330 or cBiTE = 5 ng/ml, E:T = 1:3). **B** T-cell proliferation on day 5 calculated as fold change relative to the number of T cells on day 0. **C** Percentage of T cells producing GZMB measured by flow cytometry after intracellular staining on day 5. **D** AMG 330-mediated cytotoxicity of T cells sampled at ID (*n* = 10) and RL (*n* = 7) against autologous primary AML blasts in ex vivo cytotoxicity assays (concentration AMG 330 or cBiTE = 5 ng/ml) on day 6. **E** T-cell proliferation on day 6 calculated as fold change relative to the number of T cells on day 0. **F** Percentage of CD4^+^ and CD8^+^ T cells from patients at ID (*n* = 6), RL (n = 6), and CR (n = 4) co-expressing PD-1, Tim-3, and LAG-3 on day 14 of continuous stimulation. **G** AMG 562-mediated cytotoxicity of isolated T cells against OCI-Ly1 cells after 14 days of continuous stimulation (concentration AMG 562 or cBiTE = 5 ng/ml, E:T = 1:5, 3 days). **H** Levels of secreted TNF, IFN-γ, and GZMB measured by CBA in the supernatants of cytotoxicity assays on day 3. **I** Kinetic plot and corresponding bar graphs of normalized OCR acquired during mitochondrial stress testing of T cells from patients at ID (*n* = 4), RL (*n* = 5), and CR (*n* = 4) after 14 days of continuous stimulation with AMG 562. **J** Kinetic plot and corresponding bar graphs of normalized ECAR obtained during glycolysis stress testing of T cells from patients at ID (*n* = 4), RL (*n* = 5), and CR (*n* = 4) after 14 days of continuous stimulation with AMG 562. CR complete remission; ID initial diagnosis, RL relapse. Bar plots represent the mean ± SEM. One-way ANOVA (**A**–**C** and **F**–**J**) and Mann–Whitney tests (**D**, **E**) were used to calculate *P* values.

Next, we investigated the function of BM T cells during AML progression in vitro. At ID, T cells showed lower CD3xCD33 BiTE (AMG330)-mediated cytotoxicity and T-cell proliferation against the AML cell line (OCI-AML3) relative to RL T cells (Fig. 2A–C; Supplementary Fig. S4A–C). These findings were further validated in cocultures with primary AML cells and autologous T cells; again, ID T cells showed inferior AMG330-mediated cytotoxicity and proliferation compared to RL (Fig. 2D, E, Supplementary Fig. S4D). To study the long-term function of ID and RL T cells, we used our previously established in vitro exhaustion model system, which provides continuous exposure to the CD3xCD19 BiTE (AMG 562) in the presence of the B-cell lymphoma cell line OCI-Ly1 [11]. Indeed, we observed a higher frequency of CD4^+^ and CD8^+^ T cells co-expressing PD-1, TIM-3, and LAG-3 at time of ID and RL compared to CR (Fig. 2F). Similar to the short-term stimulation, ID T cells showed lower cytotoxic function and proliferation relative to RL against OCI-Ly1 cells (Fig. 2G, Supplementary Fig. S4E). However, ID and RL T cells both showed less IFN-γ and GZMB production compared to T cells from CR (Fig. 2H). By assessing the metabolic activity, we observed that T cells at ID and RL showed lower mitochondrial respiration and glycolysis relative to CR. Interestingly, metabolic impairment was more prominent in T cells at RL, as evidenced by a significantly lower spare respiratory capacity (SRC) and glycolytic reserve (Fig. 2I, J). Together, these data show that after continuous BiTE stimulation cells at ID and RL exhibited decreased effector molecule production and impaired metabolic fitness in comparison to CR. Further examination of T-cell function using CD3 and CD28 beads revealed that the addition of CD28 co-stimulation, compared to BiTE-mediated T-cell activation, improved but did not fully rescue T-cell proliferation at ID and RL relative to CR (Supplementary Fig. S5A, B).

Taken together, our study provides insights into the dysfunctional state of BM T cells and the molecular determinants of their function during AML progression. Although ELN risk group attribution was well-balanced in our analyses (Supplementary Table 9), we acknowledge that genetic heterogeneity might still impose a bias. The impaired function of T cells during active disease, either at time of ID or RL, and their functional reinvigoration at first CR support the use of BiTE molecules in patients in CR. Although limited, the lessons from clinical trials so far in R/R AML patients have indicated a better clinical response to AMG 330 and CAR T cells preferentially in patients with low disease burden [15]. Thus, promoting BiTE molecules to consolidation, for example, after first-line therapy in patients at CR with MRD positivity, with restored T-cell function and favorable E:T ratio appears to be a better-suited scenario.

It is of high importance that clinical trials evaluating these therapies incorporate thorough biomarker studies, including BM biopsies to obtain a T-cell signature associated with response to treatment. These findings then need to be integrated into clinical trials, as it is likely that the one-size-fits-all approach does not apply.

## Supplementary information

Figure S1

Figure S2

Figure S3

Figure S4

Figure S5

Supplementary Table 1

Supplementary Table 3

Supplementary Table 5

Supplementary Table 8

Supplementary Information

## Data Availability

The RNA-seq and ATAC-seq data discussed in this publication have been deposited in the GEO database under the accession codes GSE261000 and GSE260999, respectively. The datasets generated and/or analyzed during the study are available from the corresponding author on reasonable request.
