# Supplementary figures and images for: Evolution of T-cell fitness through AML progression: enhanced bispecific T-cell engager-mediated function of bone marrow T cells at remission compared to initial diagnosis and relapse
Source: Leukemia. 2024 Aug 22;38(10):2270–5. doi: 10.1038/s41375-024-02387-4 (PMC11436353; doi:10.1038/s41375-024-02387-4)

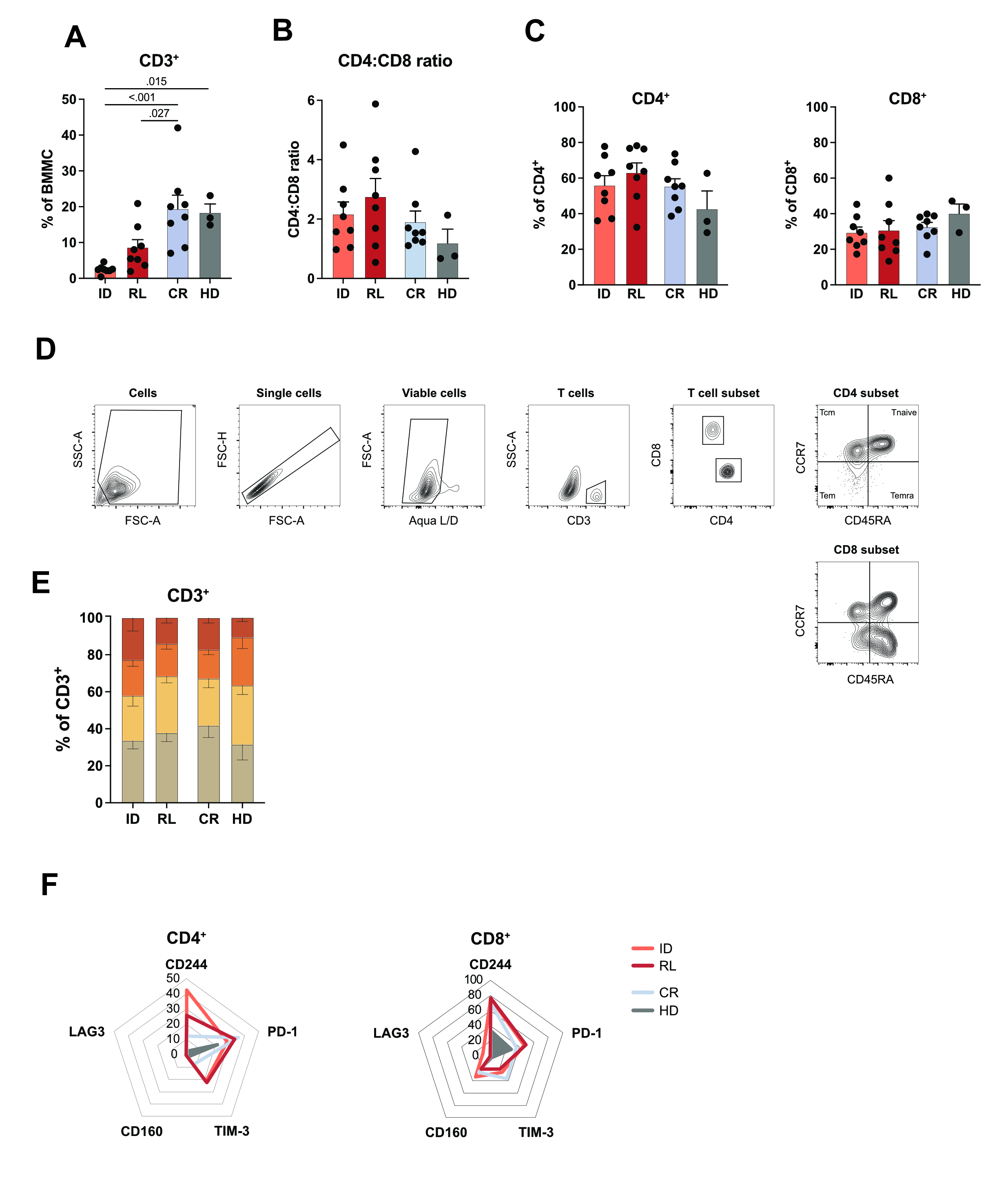

Supplement: Supplementary file 1 — Figure S1 [file 41375_2024_2387_MOESM1_ESM.tif]

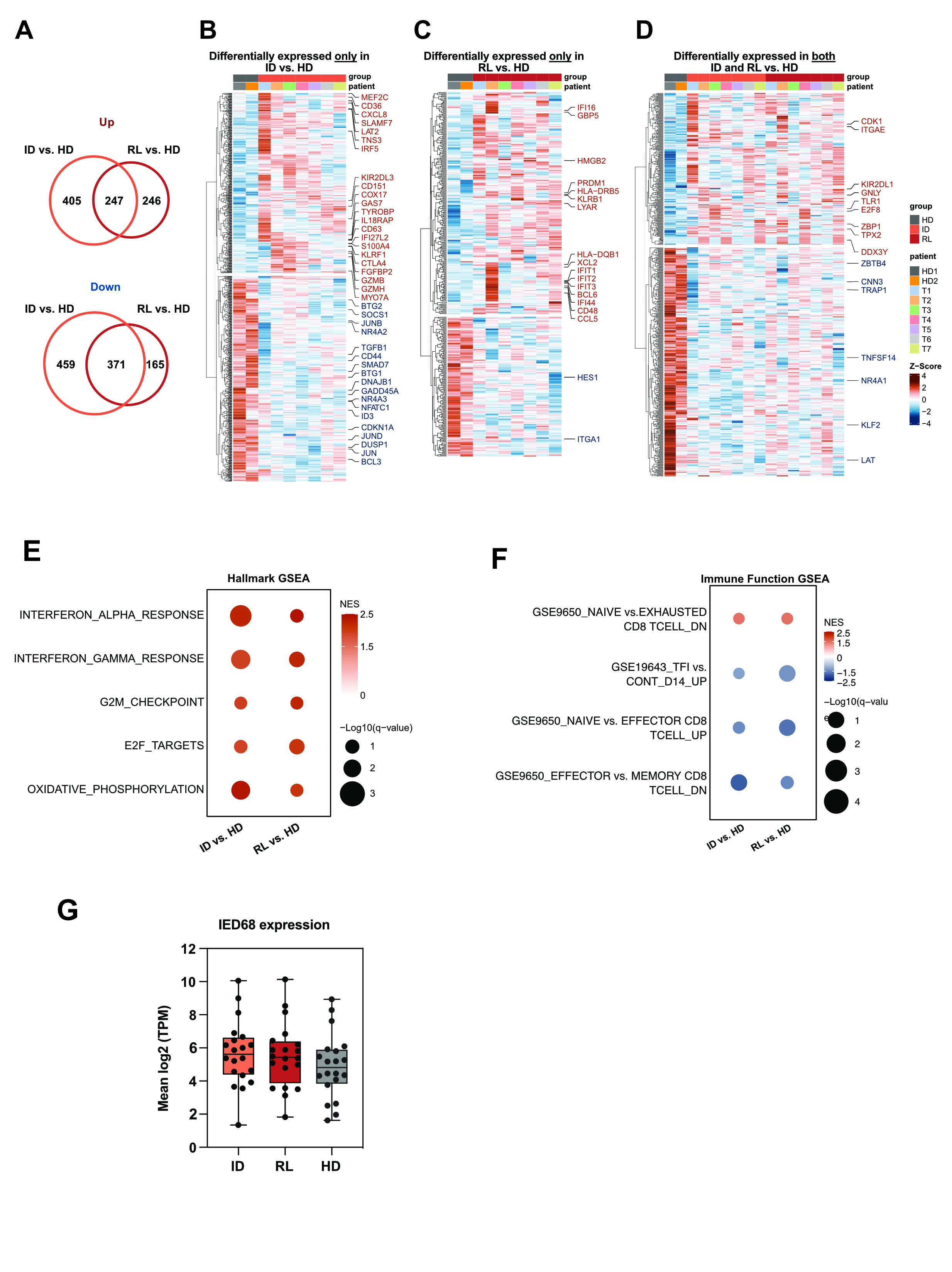

Supplement: Supplementary file 2 — Figure S2 [file 41375_2024_2387_MOESM2_ESM.tif]

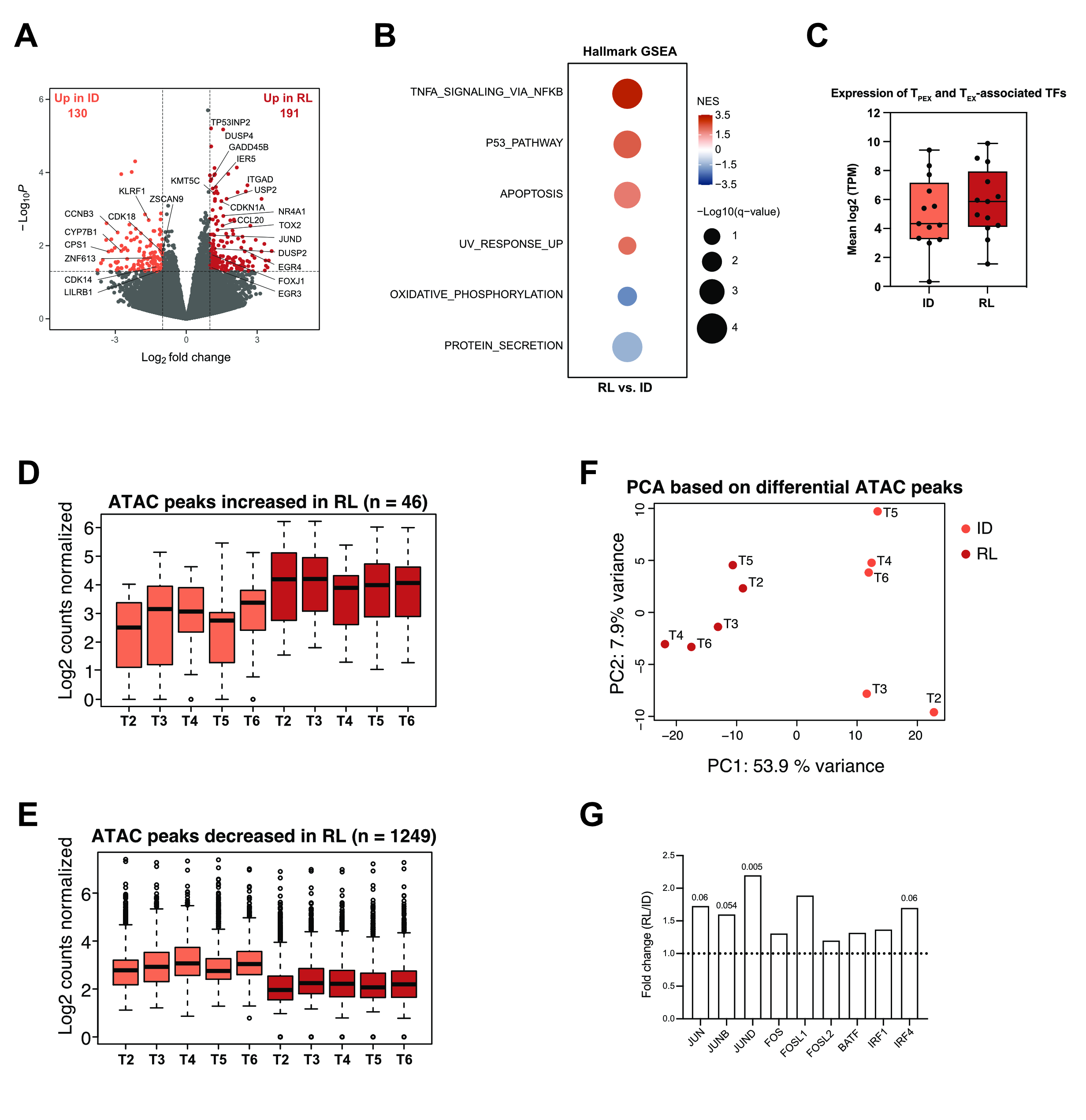

Supplement: Supplementary file 3 — Figure S3 [file 41375_2024_2387_MOESM3_ESM.tif]

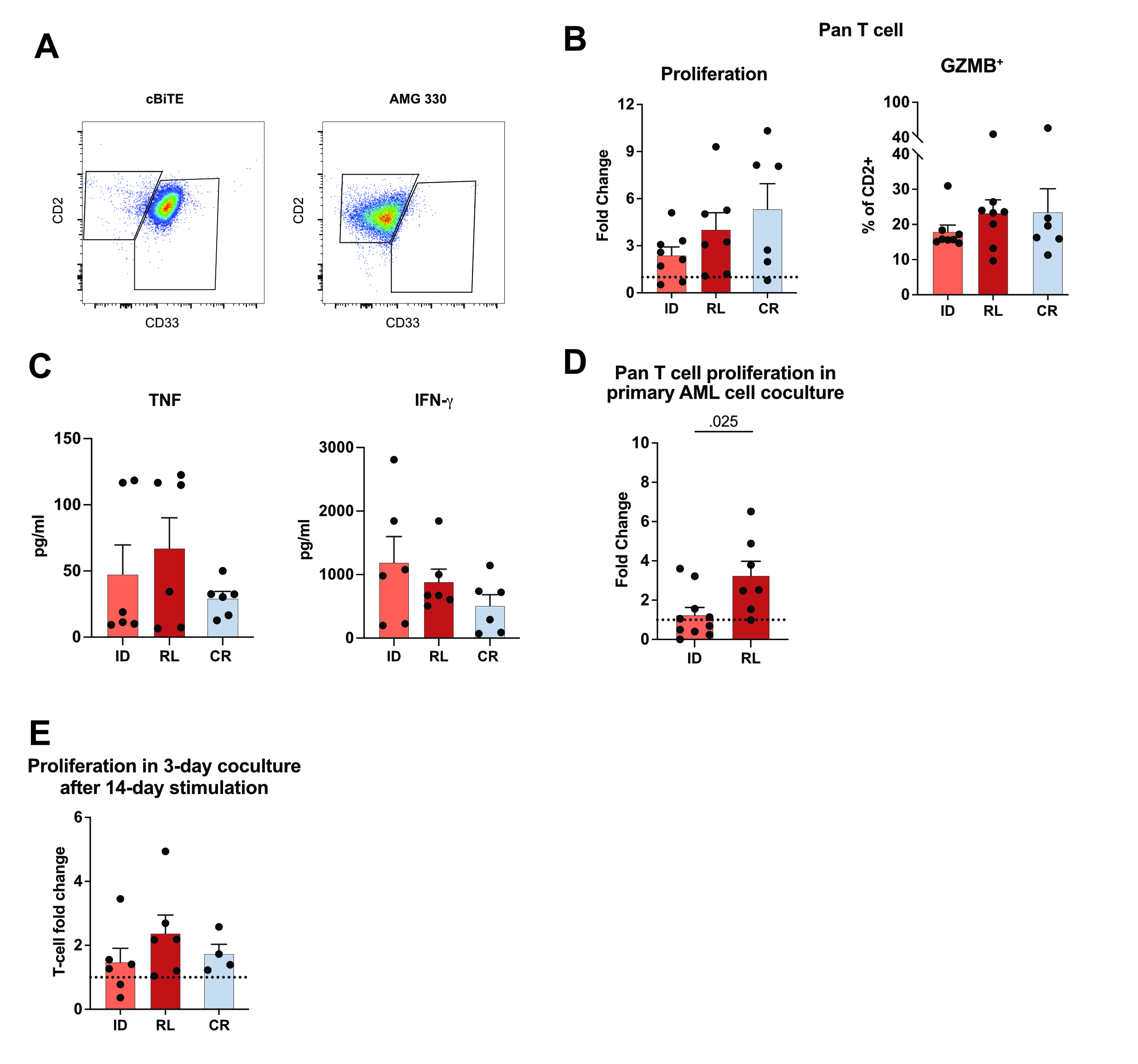

Supplement: Supplementary file 4 — Figure S4 [file 41375_2024_2387_MOESM4_ESM.tif]

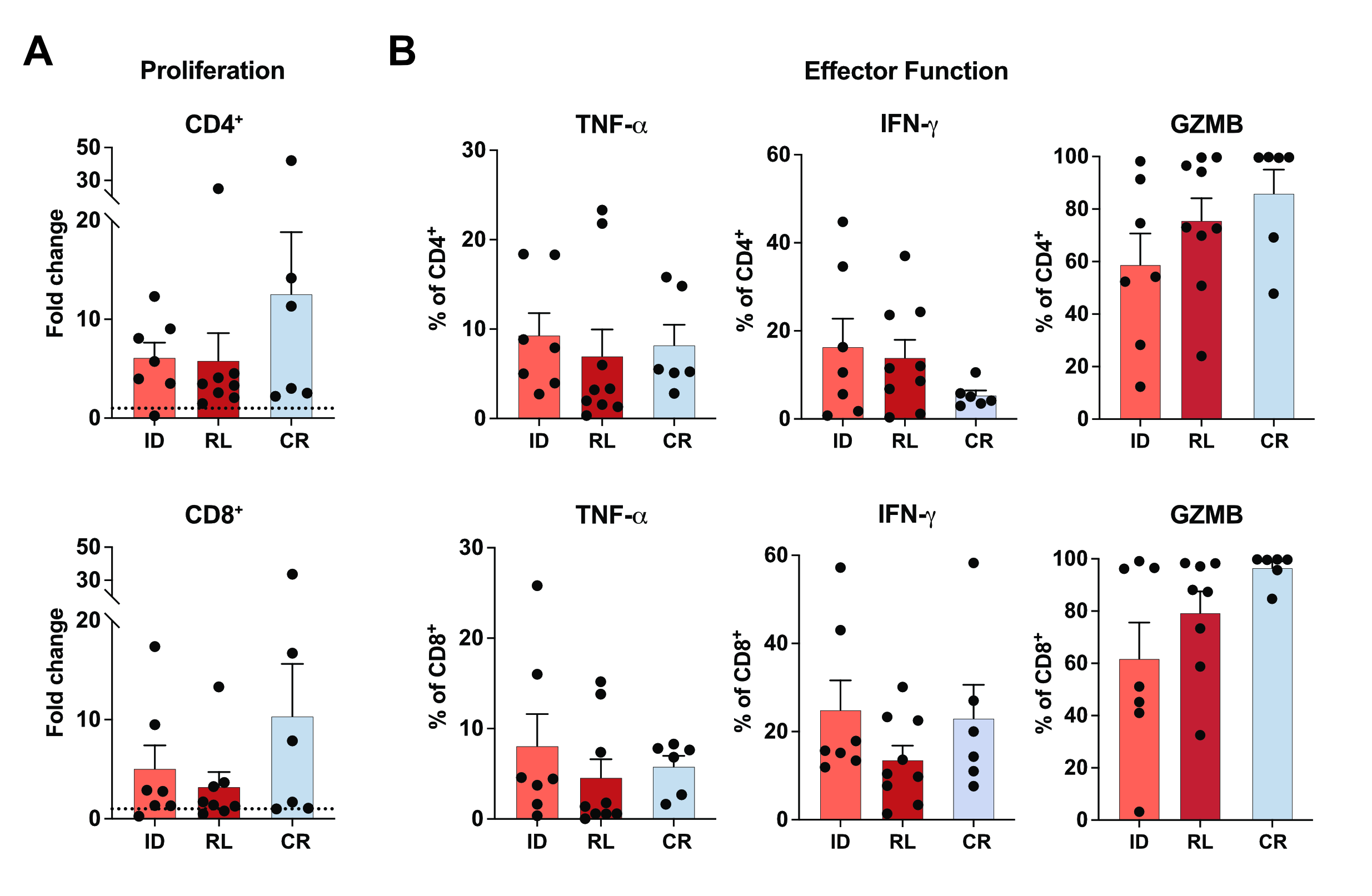

Supplement: Supplementary file 5 — Figure S5 [file 41375_2024_2387_MOESM5_ESM.tif]
