# Supplementary material for: Evolution of T-cell fitness through AML progression: enhanced bispecific T-cell engager-mediated function of bone marrow T cells at remission compared to initial diagnosis and relapse
Source: Leukemia. 2024 Aug 22;38(10):2270–5. doi: 10.1038/s41375-024-02387-4 (PMC11436353; doi:10.1038/s41375-024-02387-4)
Supplement: Supplementary file 10 — Supplementary Information [file 41375_2024_2387_MOESM10_ESM.docx]

**Supplementary Information**

**Supplementary Materials and Methods**

**Patients**

Bone marrow mononuclear cells (BMMCs) were collected from HDs (n = 15) and AML patients at ID (n = 42), RL (n = 38), and CR (n = 17) (Supplementary Table 1) giving written informed consent in accordance with the Declaration of Helsinki, and with the approval of the Institutional Review Board of the Ludwig Maximilian University (Munich, Germany). Only AML samples collected before allo-HSCT were included in this study. If applicable, patients were included in the multicenter German AML Cooperative Group (AMLCG) Registry (DRKS00020816).

**Immunophenotyping of T cells**

Immunofluorescent staining of T cells to assess T-cell subsets and expression of inhibitory receptors was performed on bone marrow mononuclear cells (BMMCs) using fluorescence-conjugated monoclonal antibodies as previously described^1^. The antibodies used and the corresponding isotype controls are listed in Supplementary Table 2. Multiparameter flow cytometry was performed on Navios or CytoFLEX S instruments (both Beckman Coulter, Krefeld, Germany). To define the state of T-cell differentiation, we assessed CCR7 and CD45RA expression (Fig. 1C, Supplementary Fig. S1B).

**Cell line**

Cell line OCI-AML3 was obtained from the German Collection of Microorganisms and Cell Cultures GmbH (DSMZ) and tested monthly for mycoplasma contamination. Authentication by STR profiling was conducted in August 2023.

**BiTE-mediated cytotoxicity assays**

*In vitro* cytotoxicity assays were performed in co-cultures of T cells from patients and OCI-AML3 cells in RPMI 1640 (PAN-Biotech, Aidenbach, Germany) supplemented with 10% FBS, 1% penicillin–streptomycin–glutamine (Thermo Fisher Scientific, Waltham, MA, USA), and 1% HEPES (Carl Roth, Karlsruhe, Germany), hereafter referred to as R10. Co-cultures were performed at an effector-to-target ratio (E:T) of 1:3 in the presence of AMG 330 or a control construct (cBiTE, both 5 ng/ml; both provided by AMGEN). After 5 days, cells were incubated for 4 hours with GolgiStop/GolgiPlug solution containing 25 nM monensin and 10 ng/ml brefeldin A (all Sigma–Aldrich, St. Louis, MO, USA) followed by staining with Aqua Live/Dead and antibodies against CD33, CD2, CD4, and CD8. Cells were then permeabilized using BD Cytofix/Cytoperm Kit (BD Biosciences, San Jose, CA, USA) and intracellularly stained for granzyme B (GZMB) or isotype control (Supplementary Table 2). Specific lysis was calculated according to the following equation: % specific lysis = [1 – (number of viable CD33^+^ target cells in AMG 330 condition)/(number of viable CD33^+^ target cells in cBiTE condition)] × 100. T-cell proliferation was determined as fold change by calculating the number of T-cells on day 5 relative to day 0. TNF-α and IFN-γ secretion was assessed in supernatants of the co-culture via cytometric bead array (CBA) using Th1/Th2 Cytokine Kit II (BD Biosciences, Franklin Lakes, NJ, USA) according to the manufacturer’s instructions.

To assess the cytotoxicity of T cells against primary AML blasts, BMMCs from primary AML samples were thawed and directly co-cultured on a feeder layer of irradiated murine MS5 stromal cells in α-MEM (PAN Biotech) supplemented with 12.5% FBS, 12.5% horse serum (Sigma–Aldrich), 1% penicillin–streptomycin–glutamine and 20 ng/ml of the recombinant human granulocyte colony-stimulating factor (rh G-CSF), interleukin 3 (rh IL-3), thrombopoietin (rh TPO; all Peprotech, Hamburg, Germany), and 57.2 µM 2-mercaptoethanol (Sigma–Aldrich). After 6 days of co-culture, the specific lysis was calculated, as described above. T-cell proliferation was calculated as fold change according to the number of T cells on day 6 relative to the number of residual T cells within the primary AML sample on day 0.

**CD3/CD28 bead stimulation assay**

T cells from AML patients were isolated from BMMCs first through depletion of CD33^+^ cells (positive selection) and then negative isolation of pan T cells according to the manufacturer’s instructions (STEMCELL Technologies, Vancouver, Canada). Isolated T cells were then cultured in R10 and stimulated with CD3/CD28 Dynabeads (Thermo Fisher Scientific) at a bead-to-T-cell ratio of 1:2 and 30 U/ml IL-2 (R&D Systems, Minneapolis, MN, USA) for 5 days. T-cell proliferation was calculated by the number of T cells relative to day 0. TNF-α, IFN-γ, and GZMB production was assessed by intracellular staining, as detailed above, with the respective antibodies or isotype controls listed in Supplementary Table 2.

**14-day stimulation of T cells with AMG 562**

To isolate T cells, BMMCs from AML patients were depleted for CD33^+^ cells (STEMCELL Technologies) and cocultured with irradiated CD19^+^ OCI-Ly1 cells in R10 at an effector-to-target ratio (E:T) of 1:4 containing 5 ng/ml AMG 562, as described previously^2^. Medium, target cells, and AMG 562 were replenished every three days. On day 14, T cells were isolated from cultures and analyzed for their phenotypic, functional, and metabolic profile, as described below.

**Immunophenotyping of continuously stimulated T cells**

On day 14 of the continuous stimulation, T cells were isolated using the pan T cell isolation kit (STEMCELL Technologies) and were stained with Aqua Live/Dead, CD2, CD4, CD8, PD-1, Tim-3, and LAG-3 antibodies.

**Annexin V staining**

Isolated T cells on day 14 of continuous stimulation were tested for apoptosis by using an Annexin V Apoptosis Detection kit (Thermo Fisher Scientific, Eugene, OR, USA) according to the manufacturer’s recommendations. Aqua Live/Dead was added to distinguish the dead cells. The frequencies of early apoptotic cells (AnnV^+^ Live/Dead^-^) and dead cells (AnnV^+^ Live/Dead^+^) apoptotic cells were determined by flow cytometry.

**AMG 562-mediated cytotoxicity assay**

T cells were co-cultured with OCI-Ly1 cells at an effector-to-target ratio (E:T) of 1:5 and 5 ng/ml AMG 562 or cBiTE. After 3 days, cells were stained with Aqua Live/Dead, CD2, CD4, CD8, and CD19 antibodies. Specific lysis and T-cell proliferation were calculated as described for other cytotoxicity assays. Levels of TNF-α, IFN-γ, and GZMB secretion in supernatants were measured using CBA.

**Metabolic stress tests**

T cells isolated on day 14 of continuous stimulation were stimulated with CD3/CD28 Dynabeads (Thermo Fisher Scientific, Waltham, MA, USA) for 48 hours, as detailed previously^2^. After bead depletion, 2.50×10^5^ T cells/well were plated on a poly-D-lysine-coated 96-well Seahorse utility plate (Agilent, Santa Clara, CA, USA). Mitochondrial and glycolysis stress tests were carried out on a Seahorse XFe96 Analyzer using corresponding kits (Agilent). Metabolic rate was normalized to cell count using a Cytation 1 reader (BioTek Instruments, Inc., Winooski, VT).

**Bulk RNA sequencing**

CD3^+^ T cells (5×10^3^) from paired ID and RL BMMCs were sorted into 100 µl of extraction buffer from a PicoPure RNA Isolation Kit (Applied Biosystems, Carlsbad, CA, USA). Total RNA was isolated according to the manufacturer’s guidelines. DNase treatment was carried out on a column using the RNase-Free DNase Set (Qiagen, Valencia, CA, USA). RNA quantity and quality was evaluated using an Agilent 2100 Bioanalyzer paired with the Agilent RNA 6000 Pico Kit (Agilent Technologies, Waldbronn, Germany). RNA samples of high quality, characterized by an RNA integrity number (RIN) > 8, were used for cDNA synthesis using the SMART-Seq v4 Ultra Low Input RNA Kit (Takara, Mountain View, CA, USA) according to the manufacturer’s protocol. Prior to library preparation, cDNA was sheared in a Covaris S220 device (PP 175; DF 10; CB 200; 5 min; 4°C) to achieve sizes ranging from 200 to 500 bp. Libraries were prepared using the MicroPlex Library Preparation Kit v2 (Diagenode, Denville, NJ, USA). Libraries were quantified using a Qubit 4.0 Fluorometer with the Qubit dsDNA HS Assay Kit (Thermo Fisher Scientific, Eugene, OR, USA). Subsequent qualification of the libraries was carried out with the Agilent High Sensitivity DNA Kit (Agilent Technologies, Waldbronn, Germany). Finally, sequencing was performed using Illumina’s HiSeq 1500 sequencer for 50 bp paired end reads.

**RNA-seq bioinformatic analysis**

Sequencing reads were aligned to release 101 of Ensembl GRCh38 prior to counting reads per gene with Star (version 2.7.6a) using default parameters. Values for expression (in transcripts per million, TPM) were calculated with RSEM (1.3.3) using default parameters. Differential expression was estimated with DESeq2 (1.28.1). Heatmap visualizations were based on log_2_-transformed TPM values. Pathway enrichment analysis was performed on ranked test statistics with the Bioconductor package "fgsea" (version 1.14.0) using the human MSigDB Collection v5p2. Gene set enrichment analysis (GSEA) for published and custom gene sets was performed with GSEA software^3^. GSEA was performed using gene sets from transcriptome analysis of ID T cells versus HD (Knaus_ID vs. HD_UP/DN) and non-responders versus responders to chemotherapy (Knaus_NR vs. CR_UP/DN)^4^, *in vitro* continuously stimulated versus treatment-free-interval (TFI)-experienced T cells (GSE196463)^2^, exhausted T cells in chronic viral infection (GSE9650)^5^, and CAR T cells generated from partial or non-responder (PR/NR) versus complete responder (CR) CLL patients (Fraietta_PR/NR vs. CR_UP)^6^. In addition, gene sets were adopted from immune effector dysfunction score (Rutella_IED68)^7^, early memory T cell (Fraietta_EARLY MEMORY T CELL)^6^, and single-cell RNA-seq of T cells (ACTIVATED CD4+_UP/DN and CD8+ TRM_UP/DN)^8^.

**Omni-ATAC-sequencing**

Assay for transposase-accessible chromatin with high-throughput sequencing (ATAC-seq) was performed as previously described^9^. Initially, 5×10^4^ CD3+ T cells from matched ID and RL BMMCs were sorted into the low-binding tubes. Cells underwent lysis in cold ATAC resuspension buffer [10 mM Tris-HCl (pH 7.4), 10 mM NaCl, 3 mM MgCl_2_] containing 0.1% NP40, 0.1% Tween-20, and 0.01% digitonin. Subsequently, nuclei were suspended in 10 μl of transposition mix, of stock of which consisted of 25 μl of 2x tagmentation buffer [20 mM Tris-HCl (pH 7.6), 10 mM MgCl_2_, 20% dimethylformamide, H_2_O], 2.5 μl of Tn5 transposase (Illumina, San Diego, CA, USA), 5.25 μl of H_2_O, 16.5 μl of PBS, 0.25 μl of 2% digitonin (Promega, Madison, WI, USA), and 0.5 μl of 10% Tween-20. The reaction was incubated for 30 minutes at 37°C with agitation at 900 rpm. DNA purification was carried out using the PCR clean-up MinElute kit (Qiagen). The transposed DNA was then amplified in 50 μl reactions using custom primers, as detailed previously^10^. The amplified libraries were purified using the PCR clean-up MinElute kit (Qiagen), with size selection for fragments of less than 600 bp achieved using Agencourt AMPure XP beads (Beckman Coulter). Libraries were quantified using a Qubit 4.0 Fluorometer paired with the Qubit dsDNA HS Assay Kit (Thermo Fisher Scientific, Eugene, OR, USA), followed by further library qualification using the Agilent High Sensitivity DNA Kit (Agilent Technologies, Waldbronn, Germany). Libraries were sequenced using Illumina’s HiSeq 1500 sequencer for 50 bp single end reads.

**Omni-ATAC-seq bioinformatic analysis**

The PEPATAC pipeline (version 0.10.3) was applied for primary analysis and quality controls with default settings^11^. In brief, after adapter trimming by Skewer^12^, reads were pre-aligned with bowtie2^13^ to human_chrM2x mitochondrial genome (parameters: -k 1 -D 20 -R 3 -N 1 -L 20 -i S,1,0.50) to remove mitochondrial reads, and subsequently mapped to the hg38 human genome (parameters: -- very-sensitive -X 2000). Following alignment, reads with low mapping quality scores (less then10) were removed and Samblaster^14^ was used to remove deduplicated reads. Peak-calling was carried out by MACS2^15^ (parameters: --shift -75 --extsize 150 --nomodel --call-summits -- nolambda --keep-dup all -p 0.01) and called peaks were filtered against the ENCODE blacklist^16^. To generate the consensus peaks matrix, overlapping peaks between every sample and the consensus peak’s coordinates based on the overlapping peak with the highest score were defined. Peaks present in at least two samples were considered. The consensus peak counts table was loaded into R (version 4.1.0) and DESeq2 (version 1.32.0) was used for normalization and identification of significant differential accessible regions (*P* value <0.05 and log_2_ fold change > 1). Motif enrichment for the peak set was performed with findmotifs.pl in Homer.

**Data visualization and statistical analysis**

Acquired flow cytometry data were analyzed using FlowJo software version 10.5.2 (Tree Star Inc., Ashland, OR, USA). Prism 9 version 9.2.0 (283) (GraphPad Software Inc.) was used for data visualization and statistical analysis. All data points were included in the analysis. A one-way ANOVA with post-hoc Tukey's correction for multiple comparisons was used to calculate the *P* values. RNA-seq and ATAC-seq data were visualized in R version 4.3.0.

For the original data, please contact Marion.Subklewe@med.uni-muenchen.de.

**Code Availability**

All custom R codes are available on request.

**Supplementary Figure Legends**

**Figure S1. Phenotypic characterization of BM T cells**

**A)** Frequency of CD3^+^ T cells in the BM of AML patients (n = 8 for all time points) and HDs (n = 3). **B)** The ratio of CD4^+^ to CD8^+^ T cells in the BM of AML patients and HDs. **C)** Frequency of CD4^+^ and CD8^+^ T cells in the BM of AML patients (n = 8 for all time points) and HDs (n = 3). **D)** Representative gating strategy for identifying T-cell subsets. **E)** Proportions of naive (T_Naive_, CD45RA^+^CCR7^+^), central memory (T_CM_, CD45RA^−^CCR7^+^), effector memory (T_EM_, CD45RA^−^CCR7^−^), and terminal effector (T_EMRA_, CD45RA^+^CCR7^−^) T cells within the CD3^+^ compartments. **F)** Spider plots showing the mean percentages of CD4^+^ (left) and CD8^+^ (right) T cells expressing inhibitory receptors. BM: bone marrow; HD: healthy donor; All plots represent the mean ± SEM. One-way ANOVA was used to calculate *P* values.

**Figure S2. Transcriptional changes in T cells from AML patients**

**A)** Scaled Venn diagram indicating the genes significantly upregulated (top) and downregulated (bottom) uniquely either at ID or RL and those common to both ID and RL, in comparison to HDs. **B)** Heatmap showing the genes differentially expressed only at ID, compared to HDs. Selected genes are highlighted. **C)** Heatmap indicating the genes differentially expressed only at RL, compared to HDs. **D)** Heatmap showing the genes differentially expressed at both ID and RL, compared to HDs. **E)** GSEA for hallmark gene signatures derived from MSigDB using GSEA software (Broad Institute). **F)** GSEA for gene sets associated with T-cell populations and exhaustion from published data sets (details on the gene sets are provided in Supplementary Methods). GSEA statistics are included in Supplementary Table 4. **G)** Mean expression of the leading-edge genes from GSEA for IED68 gene set in T cells from AML patients and HDs (related to GSEA in Fig. 1E). GSEA: gene set enrichment analysis; HD: healthy donor; ID: initial diagnosis; RL: relapse.

**Figure S3. Transcriptional and epigenetic changes in RL T cell relative to ID**

**A)** Volcano plot of genes expressed differentially between paired ID and RL (n = 7) CD3^+^ T cells. Genes significantly upregulated at ID and RL are highlighted (log_2_FC > 1 or < −1; *P* < 0.05). Selected genes are labeled. **B)** GSEA in RL vs. ID T cells for hallmark gene signatures derived from MSigDB using GSEA software (Broad Institute). Positive enrichment is shown in red and negative enrichment is shown in blue. **C)** Mean expression of the leading-edge genes from GSEA for T_PEX_- and T_EX_-associated TFs in ID and RL T cells (related to Fig. 1H and I). **D)** Increased ATAC peaks in RL T cells compared to ID from five paired samples. **E)** ATAC peaks decreased at RL relative to ID. **F)** Principal component analysis based on the differentially accessible regions. **G)** Expression of the members of the AP1 and IRF families of TFs, indicated as fold change at RL relative to ID. GSEA: gene set enrichment analysis; ID: initial diagnosis; RL: relapse; TF: transcription factor.

**Figure S4. Gating strategy, cytokine production, and proliferation of T cells upon BiTE-mediated stimulation**

**A)** Representative gating strategy for assessing BiTE-mediated cytotoxicity. **B)** T-cell proliferation on day 5 calculated as fold change relative to the number of T cells on day 0 (left) and percentage of T cells producing GZMB measured by flow cytometry after intracellular staining on day 5 (right). **C)** Levels of secreted TNF and IFN-γ measured by CBA in the supernatant on day 5 of AMG 330-mediated cytotoxicity of T cells sampled at ID (n = 6), RL (n = 6), and CR (n = 6). **D)** T-cell proliferation on day 6 calculated as fold change relative to the number of T cells on day 0 in primary AML cell coculture. **E)** T-cell proliferation in AMG 562-mediated cytotoxicity assay on day 3 calculated as fold change relative to the number of T cells on day 0 (related to Fig 2G) ID (n = 6), RL (n = 6), and CR (n = 4). CR: complete remission; ID: initial diagnosis; RL: relapse.

**Figure S5. T cells at ID and RL have lower proliferation capacity than T cells at CR after stimulation with CD3/CD28 beads**

**A)** Proliferation of CD4^+^ (top) and CD8^+^ (bottom) T cells from patients at ID (n = 7), RL (n = 8), and CR (n = 6) on day 5 of stimulation with CD3/CD28 beads (bead-to-cell ratio = 1:2) and IL-2 (30 U/ml). T-cell proliferation on day 5 was calculated as fold change relative to the number of T cells on day 0. **B)** Percentage of CD4^+^ (top row) and CD8^+^ (bottom row) T cells producing TNF-α, IFN-γ, and GZMB analyzed by flow cytometry after intracellular staining on day 5. CR: complete remission; ID: initial diagnosis; RL: relapse. Bar plots represent the mean ± SEM. One-way ANOVA was used to calculate *P* values.

**Supplementary Tables**

**Supplementary Table 1. Patients characteristics.**

Supplementary Table 1 is provided as a separate Excel file “Supplementary Table 1 - Patients characteristics.xlsx”. The table lists clinical characteristics of the AML patients in the study.

**Supplementary Table 2. Antibodies used for flow cytometry.**

| **Antibody** | **Clone** | **Fluorochrome** | **Provider** | **Cat. #** |
| --- | --- | --- | --- | --- |
| CD2 | REA972 | APC | Miltenyi | 130-116-150 |
| CD2 | RPA-2.10 | FITC | BioLegend | 300206 |
| CD2 | RPA-2.10 | PE | BioLegend | 300208 |
| CD3 | HIT3a | FITC | BioLegend | 300306 |
| CD4 | REA623 | APC-Vio770 | Miltenyi | 130-113-223 |
| CD4 | RPA-T4 | APC-H7 | BD Pharmingen | 560158 |
| CD4 | OKT4 | PerCP-Cy5.5 | BioLegend | 317428 |
| CD8 | SK1 | APC-Cy7 | BioLegend | 344714 |
| CD8 | B9.11 | PC7 | Beckman Coulter | 737661 |
| CD8 | REA734 | VioBlue | Miltenyi | 130-110-683 |
| CD33 | WM53 | APC | BioLegend | 303408 |
| CD33 | WM53 | PerCp-Cy5.5 | BioLegend | 303414 |
| CD45 | J33 | KrO | Beckman Coulter | B36294 |
| CD45RA | HI100 | BV421 | BioLegend | 304130 |
| CD160 | 688327 | APC | R&D Systems | FAB6700A |
| CD197 (CCR7) | G043H7 | PE | BioLegend | 353204 |
| CD223 (LAG-3) | Polyclonal | APC | R&D Systems | FAB2319A |
| CD244 | C1.7 | PE | BioLegend | 329508 |
| CD279 (PD-1) | EH12.2H7 | Brilliant Violet421 | BioLegend | 329920 |
| Tim-3 | 344823 | PE | R&D Systems | FAB2365P |
| Granzyme B | GB11 | FITC | BioLegend | 515403 |
| TNFa | MAb11 | BV421 | BioLegend | 502932 |
| IFNg | B27 | PE | BioLegend | 506507 |
| AquaLiveDead | – | – | Thermo Fisher Scientific | L34957 |

**Supplementary Table 3. Differentially expressed genes in AML T cells vs. HD.**

Supplementary Table 3 is provided as a separate Excel file “Supplementary Table 3 - DEGs in AML T cells vs. HD.xlsx”. The table lists genes differentially expressed (log_2_FC > 1 or < −1 and *P* < .05) in ID and RL T cells compared to HD T cells. HD: healthy donor; ID: initial diagnosis; RL: relapse.

**Supplementary Table 4. GSEA of RNA-seq in AML T cells vs. HD.**

|  | ***ID vs. HD*** | | | ***RL vs. HD*** | | |
| --- | --- | --- | --- | --- | --- | --- |
| ***Hallmark gene sets*** | **NES** | **FDR q value** | **–log_10_ q value** | **NES** | **FDR q value** | **–log_10_ q value** |
| INTERFERON_ALPHA_RESPONSE | 2.2 | 0.003381399 | 2.470903644 | 2.4 | 0.04119084 | 1.385199351 |
| INTERFERON_GAMMA_RESPONSE | 1.94 | 0.006762797 | 2.169873649 | 2.2 | 0.020943947 | 1.67894147 |
| OXIDATIVE_PHOSPHORYLATION | 2.39 | 0.007604981 | 2.118901867 | 2.06 | 0.04798545 | 1.318890428 |
| E2F_TARGETS | 1.9 | 0.041638248 | 1.380507552 | 2.06 | 0.022594927 | 1.645989057 |
| G2M_CHECKPOINT | 1.96 | 0.051273387 | 1.290107993 | 2.2 | 0.04948987 | 1.305483687 |
| ***Immune function gene sets*** | **NES** | **FDR q value** | **–log_10_ q value** | **NES** | **FDR q value** | **–log_10_ q value** |
| Knaus_NR vs. CR_UP | 2.46 | 1.00E-04 | 4 | 2.22 | 1.00E-04 | 4 |
| Knaus_ID vs. HD_UP | 2.45 | 1.00E-04 | 4 | 1.56 | 0.013083331 | 1.883281671 |
| GSE19643_TFI vs. CONT_D14_DN | 1.99 | 1.00E-04 | 4 | 2.04 | 1.00E-04 | 4 |
| GSE9650_EXHAUSTED vs. MEMORY CD8 TCELL_UP | 1.67 | 0.01082929 | 1.965400016 | 1.54 | 0.029114166 | 1.535895646 |
| IED68 | 1.59 | 0.03397341 | 1.46886086 | 1.36 | 0.098143235 | 1.008139631 |
| GSE9650_NAIVE vs.EXHAUSTED CD8 TCELL_DN | 1.46 | 0.05194912 | 1.284421805 | 1.46 | 0.03522266 | 1.453177849 |
| GSE9650_EFFECTOR vs. EXHAUSTED CD8 TCELL_DN | 1.17 | 0.26136255 | 0.582756641 | 1.4 | 0.044277195 | 1.3538199 |
| GSE19643_TFI vs. CONT_D14_UP | -1.22 | 0.052971575 | 1.275957114 | -1.35 | 1.00E-04 | 4 |
| GSE9650_NAIVE vs. EFFECTOR CD8 TCELL_UP | -1.44 | 0.018990055 | 1.721473777 | -1.66 | 1.00E-04 | 4 |
| GSE9650_EFFECTOR vs. MEMORY CD8 TCELL_DN | -1.86 | 1.00E-04 | 4 | -1.47 | 0.006521739 | 2.185636612 |
| Fraietta_EARLY MEMORY T CELL | -2.04 | 0.006481482 | 2.188325701 | -2.3 | 1.00E-04 | 4 |
| Knaus_ID vs. HD_DN | -1.95 | 1.00E-04 | 4 | -1.79 | 1.00E-04 | 4 |

HD: healthy donor; ID: initial diagnosis; NES: normalized enrichment score; RL: relapse.

**Supplementary Table 5. Differentially expressed genes in RL T cells vs. ID**

Supplementary Table 5 is provided as a separate Excel file “Supplementary Table 5 - DEGs in RL T cells vs. ID.xlsx”. The table lists genes differentially expressed (log_2_FC > 1 or < −1 and *P* < .05) in ID T cells compared to RL T cells. ID: initial diagnosis; RL: relapse.

**Supplementary Table 6. GSEA of RNA-seq in RL T cells vs. ID**

|  | ***RL vs. ID*** | | |
| --- | --- | --- | --- |
| ***Hallmark gene sets*** | **NES** | **FDR q value** | **–log_10_ q value** |
| TNFA_SIGNALING_VIA_NFKB | 3.2 | 1.00E-04 | 4 |
| P53_PATHWAY | 2.32 | 3.00E-04 | 3.522878745 |
| APOPTOSIS | 1.92 | 5.00E-04 | 3.301029996 |
| UV_RESPONSE_UP | 2.09 | 0.023 | 1.638272164 |
| OXIDATIVE_PHOSPHORYLATION | -2.04 | 0.013 | 1.886056648 |
| PROTEIN_SECRETION | -1.45 | 1.00E-04 | 4 |
| ***Immune function gene sets*** | **NES** | **FDR q value** | **–log10 q-value** |
| CD8+ TRM_UP | 2.66 | 1.00E-04 | 4 |
| ACTIVATED CD4+_UP | 2.23 | 1.00E-04 | 4 |
| Fraietta_PR/NR vs. CR_UP | 1.87 | 1.00E-04 | 4 |
| GSE9650_EFFECTOR vs. MEMORY CD8 TCELL_DN | 1.4 | 0.002457002 | 2.609594419 |
| GSE196463_TFI vs. CONT_D14_DN | 1.4 | 0.004901961 | 2.309630148 |
| GSE23321_CENTRAL MEMORY vs. NAIVE CD8 TCELL_UP | 1.37 | 0.010256411 | 1.989004584 |
| GSE9650_NAIVE vs. EXHAUSTED CD8 TCELL_DN | 1.34 | 0.018823529 | 1.725298953 |
| GSE23321_CENTRAL vs. EFFECTOR MEMORY CD8 TCELL_UP | 1.3 | 0.032258064 | 1.491361701 |
| ACTIVATED CD4+_DN | -1.48 | 0.01 | 2 |
| CD8+ TRM_DN | -2.1 | 1.00E-04 | 4 |

HD: healthy donor; ID: initial diagnosis; NES: normalized enrichment score; RL: relapse.

**Supplementary Table 7. List of enriched T_pex_ and T_ex_ TFs in GSEA**

| **NAME** | **SYMBOL** | **TITLE** | **RANK IN GENE LIST** | **RANK METRIC SCORE** | **RUNNING ES** | **CORE ENRICHMENT?** |
| --- | --- | --- | --- | --- | --- | --- |
| row_0 | NR4A1 | nuclear receptor subfamily 4 group A member 1 [Source:HGNC Symbol;Acc:HGNC:7980] | 388 | 1.568546653 | 0.15771315 | Yes |
| row_1 | TOX2 | TOX high mobility group box family member 2 [Source:HGNC Symbol;Acc:HGNC:16095] | 686 | 1.325780153 | 0.29212075 | Yes |
| row_2 | BCL6 | BCL6 transcription repressor [Source:HGNC Symbol;Acc:HGNC:1001] | 1641 | 0.909928143 | 0.35760945 | Yes |
| row_3 | NR4A2 | nuclear receptor subfamily 4 group A member 2 [Source:HGNC Symbol;Acc:HGNC:7981] | 1863 | 0.853561223 | 0.44308096 | Yes |
| row_4 | ID3 | inhibitor of DNA binding 3, HLH protein [Source:HGNC Symbol;Acc:HGNC:5362] | 1939 | 0.839560151 | 0.5322293 | Yes |
| row_5 | TBX21 | T-box transcription factor 21 [Source:HGNC Symbol;Acc:HGNC:11599] | 2086 | 0.804054618 | 0.6149617 | Yes |
| row_6 | IRF4 | interferon regulatory factor 4 [Source:HGNC Symbol;Acc:HGNC:6119] | 2385 | 0.739880145 | 0.68525296 | Yes |
| row_7 | EOMES | eomesodermin [Source:HGNC Symbol;Acc:HGNC:3372] | 3661 | 0.539221644 | 0.69874626 | Yes |
| row_8 | FOXO1 | forkhead box O1 [Source:HGNC Symbol;Acc:HGNC:3819] | 4109 | 0.48277241 | 0.7356022 | Yes |
| row_9 | IRF1 | interferon regulatory factor 1 [Source:HGNC Symbol;Acc:HGNC:6116] | 4492 | 0.450181663 | 0.77121234 | Yes |
| row_10 | BATF | basic leucine zipper ATF-like transcription factor [Source:HGNC Symbol;Acc:HGNC:958] | 5181 | 0.39401108 | 0.78976333 | Yes |
| row_11 | TCF7 | transcription factor 7 [Source:HGNC Symbol;Acc:HGNC:11639] | 10925 | 0.106154762 | 0.5965079 | No |
| row_12 | TOX | thymocyte selection associated high mobility group box [Source:HGNC Symbol;Acc:HGNC:18988] | 17051 | -0.129514351 | 0.39218056 | No |

GSEA: gene set enrichment analysis; T_pex_: precursor exhausted T cell; TF: transcription factor; T_ex_: exhausted T cell.

**Supplementary Table 8. List of Homer TF motifs enriched in RL T cells.**

Supplementary Table 8 is provided as a separate Excel file “Supplementary Table 8 - List of homer TF motifs enriched in RL T cells.xlsx”. The table lists the TF binding motifs enriched in accessible chromatin regions in RL T cells. Motif analysis was performed in Homer. RL: relapse; TF: transcription factor.

**Supplementary Table 9. Genetic risk group distribution of the analyzed patients.**

| **Figure 1A–B, Figure S1** | **51 (100 %)** |
| --- | --- |
| Favorable | 15 (29%) |
| Intermediate | 21 (41%) |
| Adverse | 15 (29%) |
| N/A | 11 |
|  |  |
| **Figure 1C–K, Figure S2, Figure S3** | **7 (100 %)** |
| Favorable | 2 (29%) |
| Intermediate | 4 (57%) |
| Adverse | 1 (14%) |
|  |  |
| **Figure 2, Figure S4, Figure S5** | **32 (100 %)** |
| Favorable | 5 (16%) |
| Intermediate | 17 (53%) |
| Adverse | 10 (31%) |
| N/A | 7 |

**Supplementary References**

1. Schnorfeil FM, Lichtenegger FS, Emmerig K, Schlueter M, Neitz JS, Draenert R, et al. T cells are functionally not impaired in AML: increased PD-1 expression is only seen at time of relapse and correlates with a shift towards the memory T cell compartment. J Hematol Oncol. 2015;8(1):93.

2. Philipp N, Kazerani M, Nicholls A, Vick B, Wulf J, Straub T, et al. T-cell exhaustion induced by continuous bispecific molecule exposure is ameliorated by treatment-free intervals. Blood. 2022;140(10):1104–18.

3. Subramanian A, Tamayo P, Mootha VK, Mukherjee S, Ebert BL, Gillette MA, et al. Gene set enrichment analysis: A knowledge-based approach for interpreting genome-wide expression profiles. Proc Natl Acad Sci. 2005 Oct;102(43):15545–50.

4. Knaus HA, Berglund S, Hackl H, Blackford AL, Zeidner JF, Montiel-Esparza R, et al. Signatures of CD8 + T cell dysfunction in AML patients and their reversibility with response to chemotherapy. JCI Insight. 2018;3(21):e120974.

5. Wherry EJ, Ha SJ, Kaech SM, Haining WN, Sarkar S, Kalia V, et al. Molecular Signature of CD8+ T Cell Exhaustion during Chronic Viral Infection. Immunity. 2007;27(4):670–84.

6. Fraietta JA, Lacey SF, Orlando EJ, Pruteanu-Malinici I, Gohil M, Lundh S, et al. Determinants of response and resistance to CD19 chimeric antigen receptor (CAR) T cell therapy of chronic lymphocytic leukemia. Nat Med. 2018;24(5):563–71.

7. Rutella S, Vadakekolathu J, Mazziotta F, Reeder S, Yau T-O, Mukhopadhyay R, et al. Immune dysfunction signatures predict outcomes and define checkpoint blockade–unresponsive microenvironments in acute myeloid leukemia. J Clin Invest. 2022;132(21):223–223.

8. Lasry A, Nadorp B, Fornerod M, Nicolet D, Wu H, Walker CJ, et al. An inflammatory state remodels the immune microenvironment and improves risk stratification in acute myeloid leukemia. Nat Cancer. 2023;4(1):27–42.

9. Corces MR, Trevino AE, Hamilton EG, Greenside PG, Sinnott-Armstrong NA, Vesuna S, et al. An improved ATAC-seq protocol reduces background and enables interrogation of frozen tissues. Nat Methods. 2017;14(10):959–62.

10. Buenrostro JD, Giresi PG, Zaba LC, Chang HY, Greenleaf WJ. Transposition of native chromatin for fast and sensitive epigenomic profiling of open chromatin, DNA-binding proteins and nucleosome position. Nat Methods. 2013;10(12):1213–8.

11. Smith JP, Corces MR, Xu J, Reuter VP, Chang HY, Sheffield NC. PEPATAC: an optimized pipeline for ATAC-seq data analysis with serial alignments. NAR Genomics Bioinforma. Oxford Academic; 2021;3(4).

12. Jiang H, Lei R, Ding SW, Zhu S. Skewer: A fast and accurate adapter trimmer for next-generation sequencing paired-end reads. BMC Bioinformatics. 2014;15(1):1–12.

13. Langmead B, Salzberg SL. Fast gapped-read alignment with Bowtie 2. Nat Methods. 2012;9(4):357–9.

14. Faust GG, Hall IM. SAMBLASTER: fast duplicate marking and structural variant read extraction. Bioinformatics. 2014;30(17):2503–5.

15. Zhang Y, Liu T, Meyer CA, Eeckhoute J, Johnson DS, Bernstein BE, et al. Model-based analysis of ChIP-Seq (MACS). Genome Biol. 2008;9(9):1–9.

16. Amemiya HM, Kundaje A, Boyle AP. The ENCODE Blacklist: Identification of Problematic Regions of the Genome. Sci Reports. 2019;9(1):1–5.
